# Patient reported outcomes used in clinical trials and core outcome sets for individuals with genetic intellectual disability: a scoping review

**DOI:** 10.1186/s11689-025-09633-5

**Published:** 2025-07-31

**Authors:** Nadia Y. van Silfhout, Maud M. van Muilekom, Clara D. van Karnebeek, Joost G. Daams, Lotte Haverman, Agnies M. van Eeghen

**Affiliations:** 1 Department of Child and Adolescent Psychiatry & Psychosocial Care, Emma Children’s Hospital, Amsterdam UMC location University of Amsterdam, Amsterdam, The Netherlands; 2 Department of Pediatrics, Amsterdam Gastroenterology Endocrinology Metabolism, Emma Children’s Hospital, Amsterdam UMC location University of Amsterdam, Amsterdam, The Netherlands; 3 Amsterdam Public Health Research Institute, Mental Health and Personalized Medicine, Amsterdam, The Netherlands; 4 Amsterdam Reproduction & Development Research Institute, Child Development, Amsterdam, The Netherlands; 5 Emma Center for Personalized Medicine, Amsterdam UMC, Amsterdam, The Netherlands; 6 Department of Human Genetics, Amsterdam UMC, Amsterdam, The Netherlands; 7 Medical Library, Research Support, Amsterdam UMC location University of Amsterdam, Amsterdam, The Netherlands; 8 Amsterdam Public Health Research Institute, Mental Health and Digital Health, Amsterdam, The Netherlands; 9 Amsterdam Public Health Research Institute, Aging & Later life and Personalized Medicine, Amsterdam, The Netherlands; 10 Advisium’s Heeren Loo, Amersfoort, The Netherlands

**Keywords:** Genetic intellectual disability, Patient reported outcomes, Patient reported outcome measures

## Abstract

**Background:**

The impact of genetic intellectual disability (GID) on daily life is significant. To better understand the impact of GID, it is essential to measure relevant patient reported outcomes (PROs). The aim of this study is to provide an overview of PROs used for individuals with GID, laying the groundwork for a future generic core PRO set for GID.

**Methods:**

To identify PROs used for individuals with GID, results of two literature reviews were integrated; (1) PROs extracted from a scoping review on outcomes in clinical trials, and (2) PROs identified from a scoping review on core outcome sets (COSs) for specific GIDs through a search in MEDLINE (Ovid), PsycINFO, Embase, and the COMET database. Descriptive analyses were performed.

**Results:**

In the first scoping review, 66 different PROs were identified. In the second scoping review, 22 different PROs were identified. After integrating PROs, 18 unique PROs remained, which were classified into a conceptual framework. Most frequently reported PROs were quality of life, perceived health, cognitive functioning, anxiety/stress, and depressive symptoms.

**Conclusion:**

This study provides an overview of PROs used for individuals with GID. These results will assist in developing a generic core PRO set for GID, to harmonize PROs used in care and research.

**Supplementary Information:**

The online version contains supplementary material available at 10.1186/s11689-025-09633-5.

## Background

An estimated 1–3% of the population is affected by intellectual disability (ID), defined by significant impairments in intellectual functioning and adaptive behavior, originating before the age of 18 years [1, 2]. Current techniques can now identify a genetic or other etiology in about half of the affected individuals [3], with over 1500 ID related genes now identified [4]. Individuals with ID of known and unknown etiology, henceforth collectively called genetic ID (GID), often exhibit complex physical and neuropsychiatric problems, such as congenital physical abnormalities, epilepsy, and psychiatric disorders such as autism spectrum disorders [5–7]. These clinical manifestations often occur together, with great variability within and between disorders. The diverse and often severe manifestations of GID can profoundly impact individuals’ daily lives, leading to physical and mental symptoms, and difficulties in forming friendships and engaging in activities [8–10].

Understanding how the clinical manifestations of GID impact daily life is crucial for optimal and relevant care and research. To obtain a comprehensive understanding of this impact on daily life, measuring relevant patient reported outcomes (PROs) is essential. PROs represent the perspective of patients on their health, without interference of a clinician or someone else [11]. PROs, such as pain or fatigue, are health outcomes directly reported by the patient or by a proxy, such as a caregiver, on their behalf. PROs can be multidimensional, including multiple PRO constructs (e.g., quality of life), or unidimensional, including only one specific PRO construct (e.g., anxiety). PROs provide valuable insights into how patients perceive the impact of a condition, giving researchers and clinicians a deeper and more holistic understanding of the condition. In addition, PROs can provide insight into the impact of a specific treatment on an individual’s daily functioning, showing benefits, but also revealing treatment-related side effects that may not be detectable through other clinical outcomes alone [11]. By measuring and assessing PROs, the unmet care needs of individuals with GID can be better identified, which not only assists in providing optimal care, but also in prioritizing research areas and guideline activities [12].

PROs can be measured with patient reported outcome measures (PROMs), which are standardized questionnaires completed by the patient or a proxy on behalf of the patient (e.g., caregiver). With the anticipated wave of innovative therapeutic interventions such as gene- and RNA-based therapies for individuals with GID, identifying and measuring relevant PROs with PROMs is becoming increasingly important [13]. A growing number of regulatory agencies, including the European Medicines Agency (EMA) and the Food and Drug Administration (FDA), highlight the significance of measuring PROs when evaluating the efficacy and safety of novel therapies [14–16]. Additionally, systematic monitoring of PROs in routine care can contribute to the efficiency of GID care and interventions, which are often time- and labor-intensive, especially with increasing scarcity of resources and personnel [17].

Unfortunately, due to the complex and heterogeneous manifestations of GID, selecting relevant PROs can be a challenge. As a solution, core outcome sets (COSs), including PROs, are being developed for specific GIDs, like Dravet syndrome [18]. These are henceforth referred to as condition-specific COSs. A COS is an agreed-upon, standardized set of relevant outcomes that should be measured and reported in all clinical trials for a specific condition [19], thereby facilitating the aggregation and comparison of outcomes across patient groups with the same condition [20]. However, creating over 1500 condition-specific COSs for the currently identified 1500 GIDs is neither feasible or desirable. Moreover, with potentially more than 1500 different condition-specific COSs, the aggregation and comparison of outcomes, including PROs, across various genetic subgroups will be difficult. Also, since GIDs share many comorbidities, such as epilepsy [6], sleeping disorders [21], and autism [7], this would enable harmonization of PROs, facilitating the aggregation and comparison of PRO care and research data both across and within GID groups. Therefore, the aim of this study is to provide a comprehensive overview of PROs used for individuals with GID by identifying (1) PROs used in clinical trials with individuals with GID, and (2) PROs included in condition-specific COSs. This study serves as an initial step toward the development of a generic core PRO set for GID [22].

## Methods

### Patient population

GID was defined as disorders associated with ID with a genetic etiology involving the central nervous system during childhood. Individuals with ID without known etiology were also included due to shared comorbidities with GID and grouped under the term GID. Individuals with inherited metabolic disorders (IMDs) were excluded, as these individuals fall outside the scope of the future generic core PRO set.

**Fig. 1:**
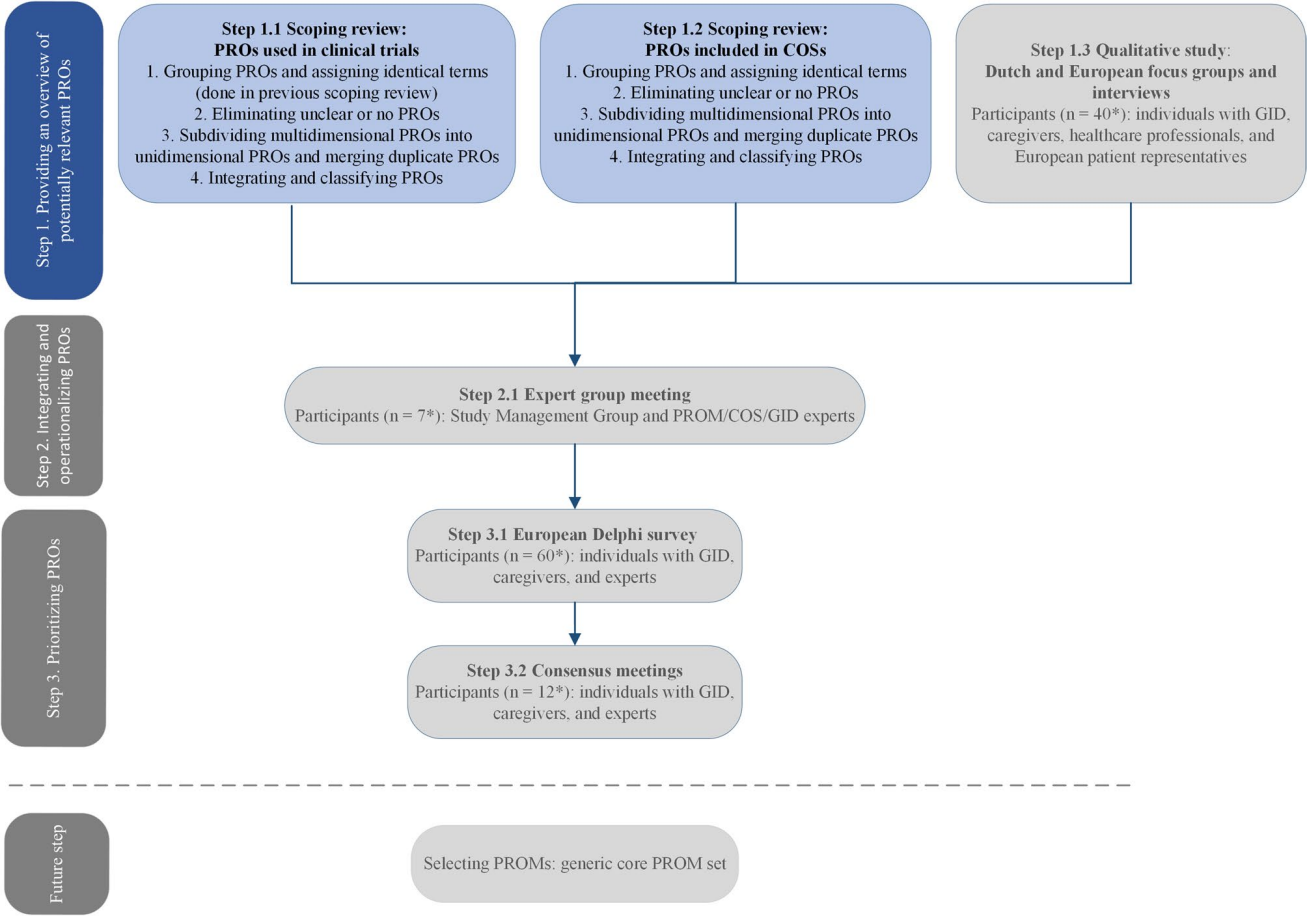
Steps for developing a generic core PRO set^1^: step 1.1 and 1.2 were undertaken in this study. ^1^Figure 1: Reproduced/adapted from van Silfhout et al. [22] under the Creative Commons license (https://creativecommons.org/licenses/by/4.0/). PROs, patient reported outcomes; PROM(s), patient reported outcome measure(s); COS(s), core outcome set(s); GID, genetic intellectual disability. *Target number of participants

To identify PROs used for individuals with GID, we integrated the results of two literature reviews, as described in Fig. 1.

### PROs used in clinical trials

To identify PROs used in clinical trials with individuals with GID, we extracted PRO data from a previous scoping review on various outcomes (i.e., clinician reported outcomes, observer reported outcomes, performance reported outcomes, and PROs) used in clinical trials with individuals with GID [23]. The methodology of the prior scoping review was aligned with the Preferred Reporting Items for Systematic Reviews and Meta-Analysis (PRISMA) Protocols and the PRISMA extension for Scoping Reviews (PRISMA-ScR) [24, 25].

#### Eligibility, search strategy, and study selection

For this scoping review, we included any clinical trial with individuals with GID measuring at least one PRO. The search was conducted in April 2022. The search strategy can be found in the original publication [23]. One team member (NvS) screened all identified studies in the original Excel database.

#### Data extraction

One team member (NvS) extracted the following data, and this was cross-checked by another team member (MvM): Title, year of publication, first author, PRO(s) measured, and dimensionality of the PRO(s) (unidimensional or multidimensional).

### PROs included in condition-specific COSs

To identify PROs included in existing condition-specific COSs, we conducted a new scoping review following the PRISMA Extension for Scoping Reviews (PRISMA-ScR) guidelines [24]. We published the protocol beforehand in the Open Science Framework (OSF): https://osf.io/nxb7t/.

#### Eligibility

COS was defined as an agreed standardized set of outcomes which should be measured and reported in all clinical trials for a specific condition. We included any type of development or use of a COS for a specific GID, for all purposes including research, clinical practice, as well as quality assessment. We included COSs covering all types of intervention, for instance surgical interventions. We also included studies that reflected the development of a COS and followed the same outcome identification steps similar to COS development studies, but were not specifically labeled as a COS. We excluded COS studies including no PROs.

#### Search strategy, study selection

We conducted a comprehensive search in November 2022 with the support of a research librarian (JD) in MEDLINE (Ovid), PsycINFO, Embase, and manually in the COMET database. We compiled a list of GID using the human phenotype ontology (HPO) database (https://hpo.jax.org/app/). We included all terms describing a genetic condition categorized under the subontology ID. Additionally, we used a search strategy for ID of unknown cause combined with terms for COS. See Additional file 1 for the full search. We identified additional studies by reference list checking. We used the application Rayyan for screening records [26]. Two members of the team (NvS, AvE) screened all titles and abstracts for potential relevance with a subsample of 10% screened for inter-rater reliability. We resolved discrepancies through discussion until consensus was achieved. Two members of the team (NvS, MvM) screened all full texts for eligibility, and discussed discrepancies until consensus was achieved.

#### Data extraction

We extracted the same data as in the first scoping review (PROs used in clinical trials). One team member (NvS) extracted the following additional data: definition of the PRO(s).

### Integrating and classifying PROs

To establish a clear set of unique PROs, we performed the following steps: all extracted PROs used in clinical trials and included in condition-specific COS were grouped together and assigned identical terms. We eliminated unclear PROs (i.e., PROs that lack clear terminology or definition) or outcomes that were not PROs. We subdivided multidimensional PROs into unidimensional PROs when feasible (i.e., multidimensional PROs consisting of distinct unidimensional PROs), and merged duplicate PROs. Subsequently, we integrated the remaining PROs identified by the two scoping reviews, and classified the PROs into a conceptual framework. This conceptual framework has been previously described in our protocol [22], and is based on the model from Valderas and Alonso [27] (combination of the model by Wilson and Cleary [28] and the International Classification of Functioning (ICF) model [29]), and the Patient-Reported Outcomes Measurement Information System (PROMIS) [30], which is further refined by PRO(M) experts in the Netherlands [31]. Within this conceptual framework, a PRO domain represents an overarching PRO (e.g., mental functioning), while a PRO subdomain reflects a specific PRO within that domain (e.g., anxiety). The conceptualization represents the definition of the PRO. We performed the classification of the PROs together with two PROM/COS experts and one GID expert (see Acknowledgements). We conducted a discussion with these experts in cases where there was uncertainty about the classification of the PROs. We also discussed the unclear PROs or outcomes that were not PROs.

## Results

### PROs used in clinical trials

Of the 317 included articles in the previous published scoping review, 213 met the inclusion criteria. An overview of the study characteristics of the included studies can be found in Additional file 2.

In total, 66 different PROs were extracted (in the previous scoping review, PROs were grouped together and assigned identical terms). After eliminating 39 PROs that did not qualify as PROs as they did not represent health aspects reported by patients (e.g., ‘behavior’, ‘self-efficacy’, or ‘social support’) or were unclear (e.g., ‘syndrome-specific symptoms’), 27 PROs remained. These were further subdivided into unidimensional PROs when feasible (e.g., ‘anxiety and depressive symptoms’ were subdivided into two unique unidimensional PROs; ‘anxiety’ and ‘depressive symptoms’), and duplicate PROs were merged, resulting in a final set of 13 unique PROs. In Table 1, the 13 unique PRO constructs are presented. The most commonly used PROs were quality of life, perceived health, cognitive functioning, anxiety/stress, and depressive symptoms.

**Table 1:** Unique pros used in clinical trials and included in five condition-specific COSs

PROs used in clinical trials			PROs included in COSs		
PRO	Frequency	%^*^	PRO	No. COS	%
Quality of life	72	34	Cognitive functioning	4	80
Perceived health	55	26	Quality of life	3	60
Cognitive functioning (attention, alertness)	47	22	Communication	2	40
Anxiety/stress	35	16	Pain (skin pain)	2	40
Depressive symptoms (suicide)	32	15	Sleep	2	40
Mental functioning	20	9	Gastrointestinal symptoms	2	40
Anger/irritability	17	8	Physical functioning	2	40
Physical (motor) functioning	16	8	Anxiety/stress	1	20
Communication	14	7	Upper extremity	1	20
Sleep	14	7	Respiratory symptoms	1	20
Participation	13	6	Mental functioning	1	20
Activity	12	6	Participation	1	20
Pain	8	4	Self-care	1	20
			Vision	1	20

*PRO* patient reported outcome, *COSs* core outcome sets

^*^Percentages are rounded

### PROs included in condition-specific COSs

**Fig. 2:**
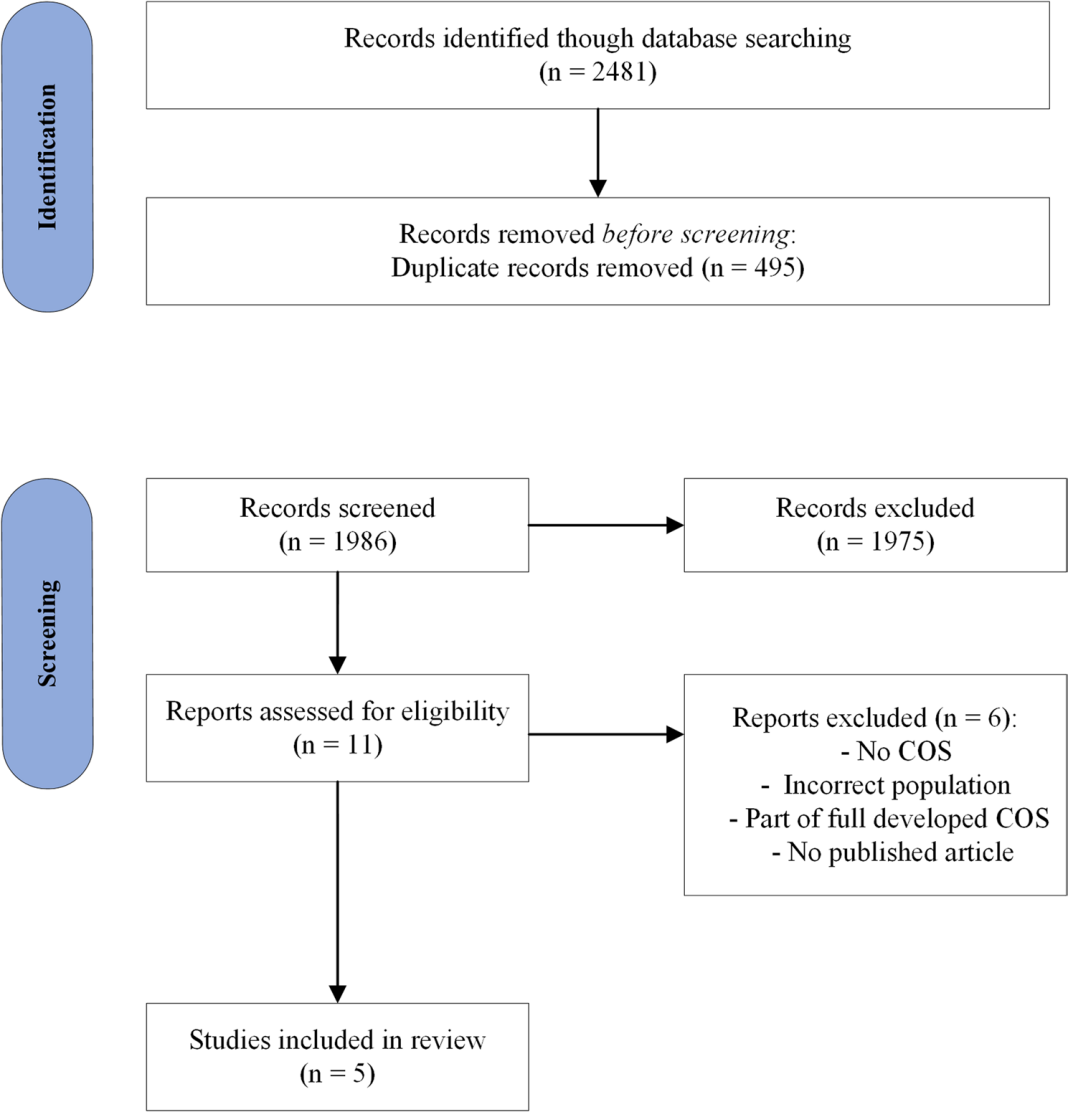
PRISMA Flowchart of the identification and selection process of condition-specific COS studies. COS, core outcome set

The literature search resulted in 1.986 articles. No additional articles were identified by reference list checking. A total of 1.975 articles were excluded based on title and abstract. Five articles met the pre-defined inclusion criteria after reviewing the full-text versions of the remaining 11 articles (Fig. 2). An overview of the study characteristics of the included studies can be found in Additional file 3.

In total, 36 PROs were extracted from the five condition-specific COSs. PROs with the same meaning were grouped together (e.g., ‘communication’ and ‘speech’) and assigned identical terms, resulting in 22 different PROs. After eliminating seven PROs that did not qualify as PROs as they did not represent health aspects reported by patients (e.g., ‘medication management’), 15 PROs remained. These were further subdivided into unidimensional PROs (e.g., ‘digestive and respiratory problems’ were subdivided into two unique unidimensional PROs; ‘gastrointestinal symptoms’ and ‘respiratory symptoms’), and duplicate PROs were merged, resulting in a final set of 14 unique PROs. Table 1 presents the 14 unique PROs. The most commonly included PROs were cognitive functioning and quality of life.

### Integrating and classifying PROs

**Fig. 3:**
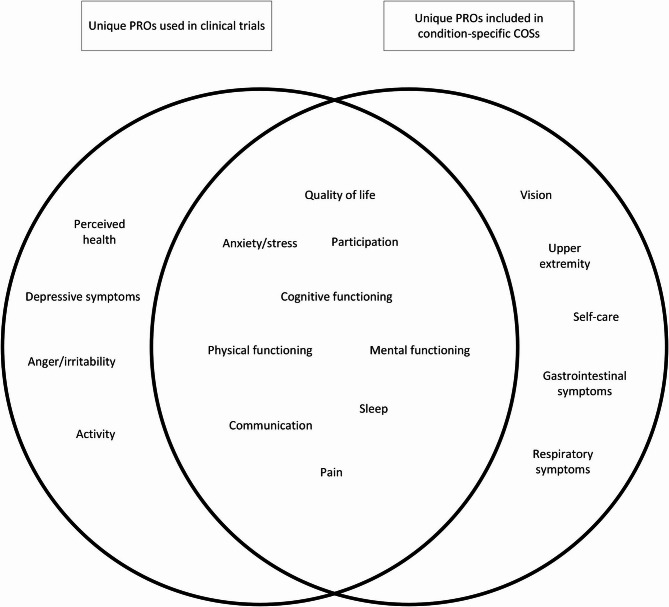
Overlapping PROs used in clinical trials and included in condition-specific COSs. PROs, patient reported outcomes; COSs, core outcome sets

The 66 different PROs identified in clinical trials, referring to 13 unique PROs, and the 22 different PROs included in condition-specific COSs, referring to 14 unique PROs, were integrated. This resulted in a total of 18 unique PROs, with 9 overlapping PROs between those used in clinical trials and those included in condition-specific COSs (Fig. 3). These 18 PROs were classified within the conceptual framework (see Additional file 4). The definition of each PRO was incorporated into the framework when described alongside the corresponding PRO in the condition-specific COSs.

## Discussion

This review provides a comprehensive overview of PROs used for individuals with GID by identifying PROs used in clinical trials and PROs included in condition-specific COSs. We identified 66 different PROs used in clinical trials, which referred to 13 unique PROs, and 22 different PROs included in condition-specific COSs, which referred to 14 unique PROs. Overlap existed between the 13 unique PROs used in clinical trials and the 14 unique PROs included in condition-specific COSs: after integrating these PROs, only 18 unique PROs remained, which were classified within a conceptual framework. The most used PROs were quality of life, perceived health, cognitive functioning, anxiety/stress, and depressive symptoms.

### Overlapping PROs

A myriad of PROs is used for GID. This diversity of PROs may arise due to the heterogeneous phenotype of GID, with a multitude of treatment targets inherent to syndromic disorders, posing a challenge on selecting relevant PROs for care and research purposes. However, even though various PROs are used for individuals with GID, considerable overlap existed between these PROs. This overlap arises due to various terms used for the same PRO (e.g., ‘communication’ and ‘speech’), and the utilization of multidimensional PROs encompassing multiple domains (e.g., ‘anxiety and depressive symptoms’). This results in an excessive number of PROs being used for GID, hampering the aggregation and comparison of PRO data both within and between various genetic subgroups.

One potential explanation for the overlap in PROs could be the presence of shared comorbidities among different GID subgroups as well, such as autism [7], epilepsy [6], and sleeping disorders [21]. These shared comorbidities may result in similar PROs, such as communication, pain, and sleep. On the other hand, it might also be possible that different clinical manifestations still lead to the same PROs. Fundamentally, all individuals desire to experience well-being and optimal functioning, such as living without symptoms (e.g., pain or fatigue), engaging in activities, and participating in society. This desire of well-being and optimal functioning can be affected by different clinical manifestations. For instance, Parkinson’s disease in chromosome 22q11.2 deletion syndrome [32], obesity in Prader-Willi syndrome [33], and early-onset dementia in Down syndrome [34] can all impact the ability to perform daily activities. Therefore, different clinical manifestations may still lead to the same PROs. This also accords with earlier studies, which showed that PROs often overlap across different conditions, such as cancer, congenital heart disease, and inflammatory bowel disease [35, 36].

### Unclear or no PROs

Some of the identified PROs were unclear (i.e., ‘syndrome-specific symptoms’), which made it difficult to know what was exactly being measured. Additionally, many of the identified PROs turned out to be no PRO at all since they did not represent health aspects reported by patients (e.g., ‘behavior’, ‘self-efficacy’, or ‘social support’). This highlights the challenge still faced by many researchers and clinicians who lack clarity on how to classify outcomes; as PROs or as other outcomes (e.g., observer reported outcomes or clinician reported outcomes).

### Need for harmonization; a generic core PRO set

This review demonstrates the need for harmonization of PROs used for GID. There is not only a myriad of PROs used for GID, but these PROs also often include different terminologies, multiple domains, and unclear definitions, making it difficult to combine or compare them properly and thus questioning their usefulness. Additionally, there still seems to be uncertainty about what constitutes a PRO and what constitutes other outcomes. Therefore, a clear and well-defined generic core PRO set is needed for GID for use in both care and research, as no such set currently exists. This generic core PRO set will ensure consistent measurement and assessment of PROs, reducing the great variety of PROs used for GID in care and research. Additionally, it will also become more clear which outcomes do not classify as PROs. This generic core PRO set may be supplemented with condition-specific PROs for particular GIDs, if necessary. The use of a generic set of PROs also aligns with the Dutch government’s initiative to measure the same PROs across conditions [31].

### Strengths and limitations

A strength of this study is the inclusion of not only clinical trials, but also condition-specific COSs, which made it possible to provide an extensive overview of PROs used for GID. A limitation of this study is that the clinical trial data is extracted from a previous published scoping review, and it may be possible that errors have occurred during the process of extraction and subsequent summarization. Moreover, because there are so few GIDs for which clinical trials exist, the yield of PROs used in clinical trials is limited and biased toward a few conditions such as Down syndrome, Fragile X syndrome, Prader-Willi syndrome, and Tuberous Sclerosis Complex. Furthermore, despite the almost 2000 hits after the literature search, only five condition-specific COSs were included. This could either mean overly strict study eligibility criteria or poor quality of study reporting. Lastly, scoping reviews have a descriptive nature and usually do not assess the quality of the included studies [37]. However, assessing the quality of the studies could enhance our understanding and assessment of the PROs found.

### Future steps

Eventually, by utilizing a generic core PRO set for GID it becomes feasible to combine and compare PRO data within and between specific GID subgroups. Additionally, it ensures the measurement of PROs that are relevant to the GID population. Therefore, further work is needed to investigate whether the PROs identified by these two scoping reviews are indeed most relevant to individuals with GID, or whether there are still potentially relevant PROs missing. Involving individuals with GID and their caregivers as both participants and co-researchers is crucial. Future steps include focus groups and interviews with individuals with GID, caregivers, and experts (e.g., healthcare professionals). After identifying all potentially relevant PROs, these PROs need to be classified within a conceptual framework, defined in detail, and then prioritized by important stakeholders, such as individuals with GID, their caregivers, and experts. Prioritizing PROs will be done through a Delphi survey and consensus meetings. The prioritization of PROs will lead to a well-defined generic core PRO set applicable to the whole GID population. Potential condition-specific PROs should also be identified and incorporated into the generic core PRO set for particular GIDs. Subsequently, suitable instruments, PROMs, must be selected to measure the generic core PRO set, which will form the generic core PROM set. This generic core PROM set needs to be validated in the GID population, and ultimately, implemented in care and research.

## Conclusions

This study offers an overview of PROs used for individuals with GID, and represents one of the first steps toward developing a generic core PRO set for GID to harmonize PROs used in care and research. The next step involves carefully selecting the instruments, PROMs, to effectively measure these PROs; the generic core PROM set. This generic core PROM set must be validated within the GID population, and implemented in care and research. Through the utilization of the generic core PROM set, combining and comparing relevant PRO data will be facilitated and continuous integration of the patient perspective into both care and research will be ensured, ultimately leading to more personalized care for individuals with GID.

## Supplementary Information

Additional file 1. Search strategy scoping review 2.

Additional file 2. Included studies in scoping review 1.

Additional file 3. Included studies in scoping review 2.

Additional file 4. Unique PROs classified within the conceptual framework.

## Data Availability

The datasets used and analyzed during the current study are available from the corresponding author on reasonable request.

## Abbreviations

ID: Intellectual disability
GID: Genetic intellectual disability
PRO: Patient reported outcome
PROM: Patient reported outcome measure
EMA: European Medicines Agency
FDA: Food and Drug Administration
COS: Core outcome set
IMDs: Inherited metabolic disorders
PRISMA-ScR: PRISMA Extension for Scoping Reviews
OSF: Open Science Framework
HPO: Human phenotype ontology
ICF: International Classification of Functioning, Disability and Health
PROMIS: Patient-Reported Outcomes Measurement Information System
